# The Role of the Immune Metabolic Prognostic Index in Patients with Non-Small Cell Lung Cancer (NSCLC) in Radiological Progression during Treatment with Nivolumab

**DOI:** 10.3390/cancers13133117

**Published:** 2021-06-22

**Authors:** Matteo Bauckneht, Carlo Genova, Giovanni Rossi, Erika Rijavec, Maria Giovanna Dal Bello, Giulia Ferrarazzo, Marco Tagliamento, Maria Isabella Donegani, Federica Biello, Silvia Chiola, Lodovica Zullo, Stefano Raffa, Francesco Lanfranchi, Giuseppe Cittadini, Cecilia Marini, Egesta Lopci, Gianmario Sambuceti, Francesco Grossi, Silvia Morbelli

**Affiliations:** 1 IRCCS Ospedale Policlinico San Martino, Nuclear Medicine, Largo Rosanna Benzi 10, 16132 Genoa, Italy; giulia.ferrarazzo@hsanmartino.it (G.F.); s4721895@studenti.unige.it (M.I.D.); silvia.chiola@humanitas.it (S.C.); s3105357@studenti.unige.it (S.R.); s4783363@studenti.unige.it (F.L.); sambuceti@unige.it (G.S.); silviadaniela.morbelli@hsanmartino.it (S.M.); 2 IRCCS Ospedale Policlinico San Martino, UOC Clinica di Oncologia Medica, Largo Rosanna Benzi 10, 16132 Genoa, Italy; carlo.genova@hsanmartino.it (C.G.); s3590678@studenti.unige.it (G.R.); mariagiovanna.dalbello@hsanmartino.it (M.G.D.B.); 3 Dipartimento di Medicina Interna e Specialità Mediche (DiMI), Facoltà di Medicina e Chirurgia, Università degli Studi di Genova, Largo Rosanna Benzi 10, 16132 Genova, Italy; marco.tagliamento@edu.unige.it; 4 Medical Oncology Unit, Fondazione IRCCS Ca’ Granda Ospedale Maggiore Policlinico, Via Francesco Sforza 28, 20122 Milan, Italy; erika.rijavec@policlinico.mi.it (E.R.); francesco.grossi@asst-settelaghi.it (F.G.); 5 Lung Cancer Unit, Medical Oncology 2, IRCCS Ospedale Policlinico San Martino, 16132 Genova, Italy; s4722270@studenti.unige.it; 6 Department of Health Sciences (DISSAL), University of Genoa, Via Antonio Pastore 1, 16132 Genoa, Italy; cecilia.marini@unige.it; 7 Department of Transitional Medicine, University of Eastern Piedmont, Via Solaroli 17, 28100 Novara, Italy; federica.biello@maggioreosp.novara.it; 8 IRCCS Ospedale Policlinico San Martino, Radiology Unit, Largo Rosanna Benzi 10, 16132 Genoa, Italy; giuseppe.cittadini@hsanmartino.it; 9 Institute of Molecular Bioimaging and Physiology (IBFM), National Research Council (CNR), Via Fratelli Cervi 93, 20090 Segrate, Italy; 10 Nuclear Medicine Unit, IRCCS Humanitas Research Hospital, Via Manzoni 56, 20089 Rozzano, Italy; egesta.lopci@humanitas.it

**Keywords:** NSCLC, immune checkpoint inhibitors, pseudoprogression, systemic inflammation index, positron emission tomography, fluorodeoxyglucose

## Abstract

**Simple Summary:**

Identifying reliable prognostic biomarkers of progression in the early phases of treatment is crucial in patients undergoing immune checkpoints inhibitors (ICI) administration for advanced non-small cell lung cancer (NSCLC). With this aim, in this study we combined the prognostic power of the degree of systemic inflammation (depicted by peripheral inflammation indexes), the quantification of the metabolically active tumor burden (estimated using 18F-fluorodeoxyglucose positron emission tomography/computed tomography) as well as their combination in NSCLC patients receiving immune checkpoints inhibitors. This combined approach could be used to improve the risk stratification and the subsequent clinical management in NSCLC patients treated with immune checkpoints inhibitors.

**Abstract:**

An emerging clinical need is represented by identifying reliable biomarkers able to discriminate between responders and non-responders among patients showing imaging progression during the administration of immune checkpoints inhibitors for advanced non-small cell lung cancer (NSCLC). In the present study, we analyzed the prognostic power of peripheral-blood systemic inflammation indexes and 18F-fluorodeoxyglucose positron emission tomography/computed tomography (FDG PET/CT) in this clinical setting. In 45 patients showing radiological progression (defined as RECIST 1.1 progressive disease) during Nivolumab administration, the following lab and imaging parameters were collected: neutrophil-to-lymphocyte ratio (NLR), derived-NLR (dNLR), lymphocyte-to-monocyte ratio (LMR), platelets-to-lymphocyte ratio (PLR), systemic inflammation index (SII), maximum standardized uptake value, metabolic tumor volume (MTV), and total lesion glycolysis (TLG). MTV and SII independently predicted OS. Their combination in the immune metabolic prognostic index (IMPI) allowed the identification of patients who might benefit from immunotherapy continuation, despite radiological progression. The combination of FDG PET/CT volumetric data with SII also approximates the immune-metabolic response with respect to baseline, providing additional independent prognostic insights. In conclusion, the degree of systemic inflammation, the quantification of the metabolically active tumor burden, and their combination might disclose the radiological progression in NSCLC patients receiving Nivolumab.

## 1. Introduction

Programmed death 1/programmed death ligand 1 (PD-1/PD-L1) inhibitors (immune checkpoints inhibitors, ICI), have been approved worldwide as therapeutic agents for immunotherapy in advanced non-small cell lung cancer (NSCLC) [1,2,3,4]. The availability of ICI has represented an essential addition to the treatment armamentarium for this population. These compounds block the interaction between PD-1 and PD-L1, sustaining the anticancer immune response with a favorable effect on disease control and overall survival (OS) [1,2,3,4]. However, in some cases, this clinical benefit is paralleled by the occurrence of atypical and challenging response patterns at computed tomography (CT) [5]. An emblematic example is represented by the so-called pseudoprogression, which is generally observed in around 3–7% of advanced NSCLC patients receiving ICI [6,7,8] and refers to disease stabilization or even an objective response following the initial disease progression.

Previous studies reported that patients showing initial radiological progression might eventually achieve a durable clinical benefit if immunotherapy is continued. In these cases, the main reason that led clinicians to continue the immunotherapy was a clinical benefit. However, treatment beyond progression seems to confer a survival benefit in only 5% of patients with asymptomatic disease progression [6,9,10]. Accordingly, the research for reliable biomarkers able to improve the response assessment in patients showing imaging progression represents an urgent need.

CT-based evaluation using the RECIST 1.1 criteria [11] is the cornerstone of imaging-based response in oncology. Given the substantial limits of size-based response in patients treated with ICI, immune-related response criteria (irRC) and immunotherapy-adapted RECIST criteria (iRECIST) have been developed [12]. One of the peculiar features of these new criteria is their ability to identify the delayed response to immunotherapy. However, to recognize the occurrence of disease progression, two consecutive follow-up imaging studies (with an interval between the assessments of at least four weeks) are needed [13]. Nevertheless, earlier identification of non-responding patients may allow stopping inappropriate treatment associated with not negligible adverse events. Further, it would also optimize costs for the healthcare system.

In the last years, it has emerged that the host’s response to the progressing malignancy significantly impacts the clinical outcomes in oncological patients [14]. In this framework, the use of circulating blood cells has been proposed as a surrogate of systemic inflammation able to provide prognostic insights in various cancer types [15,16,17,18]. As the anticancer immune response results from the complex interplay between T cells and other immune cells, peripheral biomarkers increased attention even in the setting of immunotherapy [19,20,21,22]. On the other hand, 18F-fluorodeoxyglucose positron emission tomography (FDG PET/CT) can display the tumor microenvironment’s glucose consumption [23,24,25]. Thus, it is reasonable to assume that the combination of FDG PET/CT with systemic inflammation indexes might further improve cancer patients’ prognostic stratification.

Given these premises, in the present study, we combined peripheral-blood systemic inflammation indexes and the FDG PET-based response assessment in patients showing disease progression according to RECIST 1.1 (from now on simply defined as RECIST) at CT scan assessments during treatment with ICI.

## 2. Results

### 2.1. Patients’ and Treatment Characteristics

The present study includes 45 patients receiving Nivolumab in the context of two clinical trials on ICI (registered as NCT02475382 and NCT03563482 on www.clinicaltrials.gov (accessed on the 4 June 2021), respectively) showing at least one progressive disease (PD) according to RECIST during treatment. These patients, whose clinical characteristics at the time of the radiological progression are summarized in Table 1, were included in the present analysis (*n* = 36 from NCT02475382 and *n* = 9 from NCT03563482, respectively).

All patients had a histopathological diagnosis of NSCLC. The non-squamous type was reported in 76% of cases. Previous lung surgery was performed in 38% of cases, while previous radical thoracic radiotherapy was reported only in five cases (11%). As enrolled patients were pretreated, Nivolumab was administered as second-line therapy in 18 patients (40%) and as subsequent lines in 27 (60%). At the time of radiological progression, the median age was 70.6 years (range 50.3–81.5), with eastern cooperative oncology group performance status (ECOG PS) 0–1 in 95.5% of patients. The median interval from treatment initiation and the radiological progression was three months. At the subsequent radiologic evaluation (after four weeks), according to irRC criteria, 2 cases were classified as partial response (PR, 4.5%), 7 cases with stable disease (SD, 15.5%), 18 cases with unconfirmed progressive disease (uPD, 40%), and 18 cases as confirmed (cPD, 40%). By contrast, according to iRECIST criteria, 2 (4.4%), 1 (2.2%), 33 (73.4%), and 9 (20%) cases were classified as immune-PR, immune-SD, immune-uPD, and immune-cPD, respectively.

**Figure 1:**
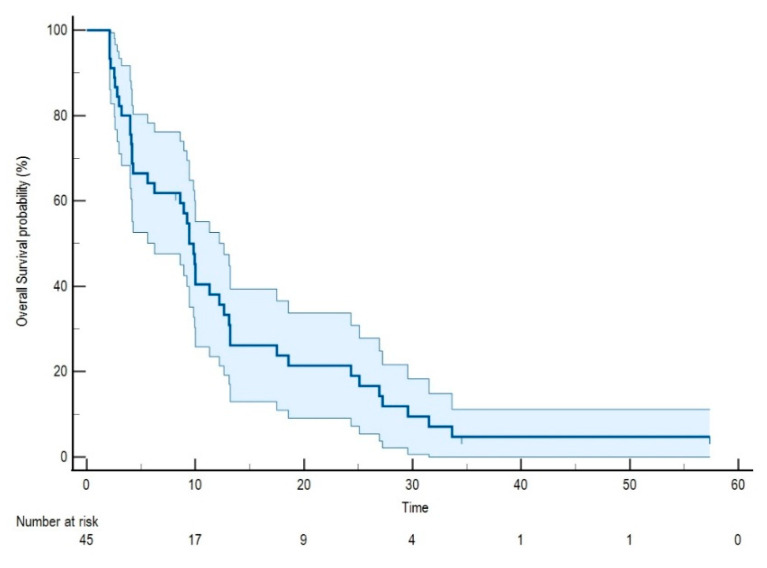
Kaplan Kaplan–Meier survival function of the study cohort.

All cases included in the study were assessable for survival analysis and were followed-up for a median interval of 9.43 months. The median OS (mOS) was 9.29 months (95% CI: 8.48–10.38). OS was 64% (95% CI: 57–71) at 6 months, while it was 38% (95% CI: 31–45) at 12 months. Figure 1 shows the Kaplan Kaplan–Meier survival function of the study cohort.

### 2.2. Systemic Inflammation Indexes and FDG-Derived Parameters at Radiological Progression

At the time of radiological progression according to RECIST criteria, maximum standardized uptake value (SUVmax), metabolic tumor volume (MTV), and total lesion glycolysis (TLG) were 12.6 ± 7.2, 208 ± 516.5, and 1040.1 ± 2788.6, respectively. According to PERCIST criteria, 33 (73.3%) were classified as progressive metabolic disease (PMD), 9 (20%) with stable metabolic disease (SMD), and 3 (6.7%) as partial metabolic response (PMR). No patient was classified as complete metabolic response (CMR). Results from the univariable Cox regression analyses are reported in Table 2. None of the included clinical characteristics reached statistical significance at univariate analysis. Among systemic inflammation indexes, neutrophil-to-lymphocyte ratio (NLR), derived-NLR (dNLR), and systemic inflammation index (SII) reached statistical significance at the univariate analysis. In all cases, higher OS was observed for lower values of these systemic inflammation parameters. Similarly, lower MTV and TLG correlated with an increased OS. Among them, only SII and MTV remained independently associated with OS at the multivariate analysis.

**Figure 2:**
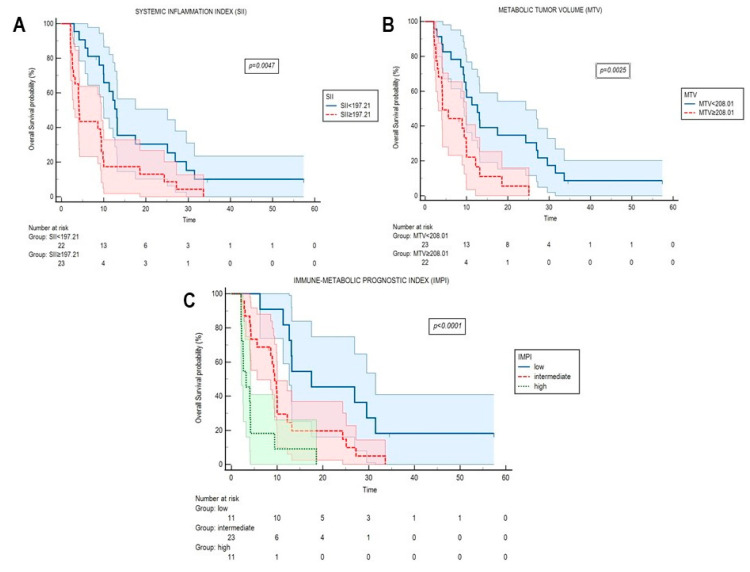
Kaplan–Meier curves for OS according to systemic inflammatory indexes, FDG-derived parameters, and their combination (IMPI) at the time of radiological progression. Kaplan–Meier curves for overall survival (OS) according to systemic inflammation index (SII, (**A**)), metabolic tumor volume (MTV, (**B**)), and their combination in the immune-metabolic prognostic index (IMPI, (**C**)). SII, MTV and IMPI were calculated at the time of radiological progression.

Results from Kaplan–Meier analyses of SII and MTV, once binarized, are reported in Figure 2A,B. The combination of the above-mentioned parameters allowed us to identify the immune-metabolic prognostic index (IMPI), which categorized the enrolled cases in three groups with different risk as follows: low (neither MTV ≥ 208.01 nor SII ≥ 197.21, IMPI = 0, *n* = 11), intermediate (MTV ≥ 208.01 or SII ≥ 197.21, IMPI = 1, *n* = 23), and high IMPI (MTV ≥ 208.01 and SII ≥ 197.21, IMPI = 2, *n* = 11). Kaplan–Meier curves for IMPI are represented in Figure 2C. Median OS was 17.5 month (95% CI 11.3–31.5 months), 9.4 months (95% CI 5.6–33.6 months), 3.2 months (95% CI 2.1–18.5 months) for the low, intermediate, and high IMPI groups, respectively (*p* < 0.0001).

### 2.3. Systemic Inflammation Indexes and FDG-Derived Parameters in the Evaluation of Response

Results from the univariable Cox regression analyses, including irRC and iRECIST, and the variation of each parameter (calculated as the ratio compared to baseline), are reported in Table 3. Apart from irRC and iRECIST criteria, only the variation of SII, SUVmax, MTV, and TLG (termed SIIratio, SUVmax-ratio, MTVratio, and TLGratio, respectively) reached significance for the prediction of OS at the univariate analyses. A lower variation of these parameters at the time of radiological progression compared to baseline was associated with a worse prognosis in all cases. In the multivariable model, irRC, SIIratio and TLGratio remained independently associated with OS.

**Figure 3:**
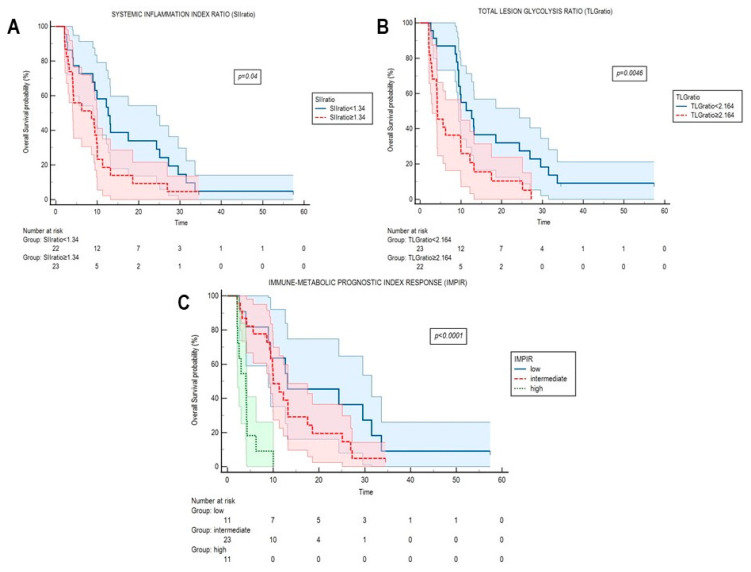
Kaplan–Meier curves for OS according to systemic inflammatory indexes, FDG-derived parameters, and their combination (IMPIR) in the evaluation of response. Kaplan–Meier curves for overall survival (OS) according to systemic inflammation index ratio (SIIratio, (**A**)), total lesion glycolysis ratio (TLG, (**B**)), and their combination in the immune-metabolic prognostic index response (IMPIR, (**C**)). SIIratio, TLGratio, and IMPIR were calculated at the time of radiological progression with respect to baseline.

Results from Kaplan–Meier analyses of these variables, once binarized, are reported in Figure 3A,B. The combination of these parameters allowed us to calculate the immune-metabolic prognostic index response (IMPIR), which identified three groups with different risk as follows: low (neither SIIratio ≥ 1.34 nor TLGratio ≥ 2.164, IMPIR = 0, *n* = 11), intermediate (SIIratio ≥ 1.34 or TLGratio ≥ 2.164, IMPIR = 1, *n* = 23), and high IMPIR (SIIratio ≥ 1.34 and TLGratio ≥ 2.164, IMPIR = 2, *n* = 11). Kaplan–Meier curves for IMPIR are represented in Figure 3C. The median OS was 13.1 months (95% CI 0.00–29.17 months), 10 months (95% CI 7.85–12.14 months), 4 months (95% CI 2.31–5.69 months) for the low, intermediate, and high IMPIR groups, respectively (*p* < 0.0001). Of note, when included in a multivariable model, IMPI and IMPIR resulted in independent predictors of OS (Table 4).

## 3. Discussion

Assessing the residual metabolically active disease burden, estimating the degree of systemic inflammation, and combining them, provide prognostic insights in advanced NSCLC showing radiological progression during ICI administration. This method may identify patients who would benefit from immunotherapy continuation, despite the radiological progression. Further, it independently stratifies OS even when its determinants are compared to baseline, suggesting its potential use as a response assessment tool.

Pseudoprogression represents an important but uncommon occurrence in the setting of ICI therapy for advanced NSCLC, and it is clinically defined when imaging shows an increase in tumor burden, despite the patient appearing clinically stable or improved. This phenomenon is thought to be a manifestation of tumor infiltration by an activated immune reaction between tumor cells and host immune cells, due to successful anti-PD1 therapy [26]. As pseudoprogression may not be fully captured by conventional CT-based response, criteria such as RECIST [23], irRC [27], and iRECIST criteria [12] have been proposed to improve and standardize response assessment in patients receiving ICI. However, both these approaches require a further CT evaluation after four weeks to rule out or confirm progression, thus delaying the interruption of ineffective therapy and adding unnecessary costs to the healthcare systems. For these reasons, the identification of alternative biomarkers potentially able to disclose responders from non-responders since the time of radiological progression has an added value in this clinical setting.

Inflammatory infiltration and related tumor changes can also hamper the FDG PET/CT-based response’s reliability. However, several studies have to date suggested a potential added value of this approach, at least in selected patients [24]. In a previous study by our group comparing the first response assessment to Nivolumab using CT and FDG PET-based criteria in the same cohort of patients, FDG imaging maintained a prognostic value even in patients classified as PD based on CT [28]. This prognostic value was also observed when tumor burden parameters (i.e., MTV and TLG) were applied [22,29,30]. Similarly, in previous studies on the use of PET-based response in melanoma patients receiving ICIs, it was demonstrated that as much as half of patients showing residual disease on CT have negative FDG-PET scans [31,32,33,34]. Even though the current literature does not support serial FDG PET/CT in the general population of NSCLC patients treated with ICIs, the present study supports the prognostic potential of the metabolic information, in specific cases, at the time of radiological progression. Moreover, it further confirms the importance of acquiring baseline FDG-PET data to compare with post-treatment examinations in complex cases, potentially improving the therapeutic decision-making.

On the other hand, in the present study, we analyzed the prognostic value of systemic inflammation indexes. Systemic inflammatory status has been closely correlated with worse prognosis in advanced NSCLC, particularly in patients treated with platinum-based chemotherapy and targeted therapies [35,36,37,38,39]. Bagley et al. [40] showed that higher baseline NLR negatively correlates with progression-free survival and OS in the immunotherapy setting. Similarly, Zer et al. [41] previously showed that lower values of NLR at baseline and during immunotherapy favorably influence indicators of treatment outcome, including disease control rate, treatment duration, time to progression, and overall survival. These findings fit with the acknowledged role of neutrophil recruitment into the tumor microenvironment. While lymphocytes are crucial in tumor defense and are associated with a favorable prognosis, neutrophils play a tumorigenic role by inhibiting apoptosis and promoting metastasis and angiogenesis [42]. Thus, the ratio between these inflammatory cells in NLR describes the balance between pro- and antitumor influences of the host microenvironment. However, the potential application of systemic inflammation indices as tools to disclose radiological progression while receiving immunotherapy is still largely unknown. To the best of our knowledge, only Kiriu et al. previously tested this approach in a series of four NSCLC patients [43], suggesting that patients with higher post-therapy NLR values should be considered for an early transition to the next drug treatment regimen. The present study extends this previous observation, comparing NLR with a high number of systemic inflammation indexes. This method allowed us to show that the absolute value of SII at the time of radiological progression overcomes the prognostic value of NLR. The same consideration applies to the variation of this parameter with respect to baseline. The added value of SII compared to other inflammation indices (including NLR) has already been demonstrated in several oncological settings [44,45,46,47], including metastatic NSCLC candidates to ICI [48]. From the pathophysiological point of view this finding is not surprising, as SII represents a composite measure of the neutrophil, lymphocyte, and platelet counts, perhaps more comprehensively describing the tumor microenvironment composition. Patients with higher SII, namely higher immune suppression in the periphery, may have a lower degree of antitumor immune response and a lower probability to show delayed shrinkage after the initial tumor growth, thus being classified as in pseudoprogression [49].

As a final consideration, in the present study, metabolic and inflammatory biomarkers were combined. While this approach has already been extensively proposed in other cancer models [50,51,52,53,54,55,56,57,58,59], to the best of our knowledge, only in the studies by Seban et al. [60,61] and Castello et al. [22], it was focused on NSCLC treated with ICI. However, while these studies demonstrated that the baseline combination of metabolic tumor burden descriptors (i.e., MTV or TLG) and inflammatory biomarkers are associated with poor OS, we focused our analysis on the radiological progression’s setting. On the one side, this combined approach allowed to simultaneously estimate the residual metabolically active disease and the degree of systemic inflammation (IMPI). On the other hand, it evaluated the metabolic and inflammatory response concerning baseline (IMPIR), which resulted prognostic even after adjusting for irRC and iRECIST classes. The obtained indexes potentially represent easy and widely applicable tools for clinical practice at no additional costs (see also Supplementary Figure S1, which summarizes how to calculate IMPI and IMPIR). Therefore, whether confirmed in larger settings, our data may help clinicians resolve doubtful cases, identify patients not benefiting from treatment with ICI, spare them from potential immune-related adverse events on one side, and reduce costs for the health system hand. Of note, IMPI and IMPIR were both potentially useful, as they independently predicted OS. Based on the present data, we cannot speculate on which metric should be preferred in the clinical setting between IMPI and IMPIR. Therefore, this point needs to be addressed by further studies.

The present study has some limitations. First, the study is based on a relatively small population, and it lacks a control group of patients who did not receive ICI. Thus, these findings should be considered preliminary, while a better-defined comparison between FDG PET/CT and peripheral inflammatory biomarkers still needs to be assessed in a larger multicentric setting. Third, the cut-off values used for inflammatory and metabolic biomarkers and their combination require further validation, as they are data driven. The unknown PD-L1 status represents the final limitation of the present study. It should be noted that enrolled patients were treated in a second- or third-line setting, in which PD-L1 status was not mandatory for prescribing ICI therapy in Europe. However, this information might improve the comprehension of obtained findings [62] and will need to be addressed by further future studies.

## 4. Materials and Methods

### 4.1. Study Population

Seventy-four patients with advanced pretreated NSCLC were enrolled in a translational research trial at the Lung Cancer Unit of the IRCCS Ospedale Policlinico San Martino. The trial was an ancillary mono-institutional study conducted within the Expaned Access Program (EAP) for Nivolumab (NCT02475382). The Ethical Committee of Regione Liguria (Italy) approved the study design. All the enrolled patients gave written informed consent to participate in this study. The inclusion criteria of the study are detailed elsewhere [28]. The major inclusion criteria were the following: age ≥18 years, histologically or cytologically confirmed NSCLC, clinical-stage IIIb or IV (according to TNM v7.0), at least one measurable lesion by RECIST 1.1; if patients had brain metastases, they had to be previously treated or stable from at least two weeks before the treatment with Nivolumab and not needing steroids with more than 10 mg/day of prednisone or equivalent. ECOG scale of performance status ≥3, meningeal carcinomatosis, active autoimmune disease or syndrome that needed daily steroids treatment (excepted for diabetes mellitus type I, hypothyroidism only requirement hormone replacement), previous line of therapy with ICI, and the administration of a life attenuated vaccine within the 30 days before the first Nivolumab administration. Baseline FDG PET/CT and CT were performed within 30 days before starting therapy with Nivolumab 3 mg/kg every 14 days. Imaging was repeated after four cycles and then every four cycles. Response to treatment was evaluated by contrast-enhanced CT and by FDG PET/CT. A flow-chart showing a schematic representation of the original study design is reported elsewhere [28].

Aiming to enlarge the study sample, data from further nine NSCLC patients treated with Nivolumab belonging to a different clinical trial for ICI (NCT03563482), meeting our inclusion criteria and showing radiological progression were considered. The details of the trial NCT03563482 are reported elsewhere [22,63,64].

### 4.2. Study Design

The present study focuses on patients experiencing radiological PD according to RECIST during treatment. Accordingly, only patients showing at least one post-therapy progression at contrast-enhanced CT (classified as PD according to RECIST criteria) were included in the analyses. According to the NCT02475382 protocol, treatment with Nivolumab was continued beyond progression in case of clinical benefit. The same consideration applies to NCT03563482. However, to allow a more direct transferability of the present findings into the clinical setting, the analyses were conducted by considering only the first radiological progression. Moreover, aiming to avoid any potential bias related to the administered treatment, patients treated with Pembrolizumab in the trial NCT03563482 were excluded. As a final inclusion criterion, only patients with available FDG PET/CT and systemic inflammation indexes at baseline and at the time of the radiological progression were selected. In the patients’ obtained subgroup, we assessed the prognostic value of FDG PET/CT-derived parameters estimating the metabolically active disease burden, systemic inflammation indexes, and their combination. The prediction of OS of their variation concerning baseline was also assessed. As detailed below, added predictive value of these parameters was adjusted for the patient class of response based on PERCIST, irRC, and iRECIST criteria [11,12,27,65].

### 4.3. Systemic Inflammation Indexes

As previously shown in the literature [66,67,68], we retrospectively collected white blood cells (WBC), platelets (PLT), absolute neutrophil (ANC), lymphocyte (ALC), and monocyte (AMC) count obtaining their ratio: NLR, derived NLR (dNLR), lymphocyte-to-monocyte ratio (LMR), platelets-to-lymphocyte ratio (PLR), and systemic inflammation index (SII). dNLR was calculated as ANC/(WBC-ANC) and SII as NLRxPLT.

### 4.4. Images Acquisition and Analysis

Images were acquired according to international guidelines as detailed elsewhere [28,69].

RECIST response was assessed by one radiologist, one oncologist, and one nuclear medicine physician experienced in response evaluation with radiological response criteria in patients treated with ICIs (G.C.; G.R.), blinded to lab and PET/CT results. CT images performed after four weeks were also analyzed in view to define the irRC and iRECIST criteria. Regarding radiologic assessments for patients receiving ICI beyond PD as assessed by RECIST, the CT scans that determined PD were used as a new “baseline”, and subsequent CT scans were compared to the new baseline according to RECIST criteria. Both PERCIST criteria and FDG PET/CT images semiquantifications were interpreted in consensus by two expert nuclear medicine physicians (M.B.; S.M.) blinded to contrast-enhanced CT. Response criteria are detailed elsewhere [11,12,27,65]. It should be noted that each post-therapy FDG PET/CT scan that was performed at the time of CT-based progression was independently compared with baseline and that the nuclear medicine physicians were also blinded to the results of previous or following 18F-FDG PET/CT examinations of the same patient. The SUVmax of the hottest lesion was obtained in the transaxial view. Further, a volume of interest was drawn using an SUV-based automated contouring program (Syngo Siemens workstation, Siemens Medical Solutions, USA) with an isocounter threshold based on 40% of the SUVmax, as previously recommended [70]. The sum of all lesions obtained MTV. TLG was calculated as the sum of the products between MTV and the corresponding SUVmean calculated within each MTV.

### 4.5. Statistical Analysis

To assess the association between systemic inflammation indexes and FDG PET/CT-derived parameters at the time of radiological progression with OS, univariable Cox regression models were used. The failure event for OS was defined as death due to any cause. Survival time was measured from the date of ICI initiation to the date of death or last follow-up. To further understand these parameters’ prognostic values, continuous variables were binarized using the median value was used as a cut-off. Univariate OS curves were then computed according to Kaplan–Meier and compared with the Log Rank test. All parameters, biomarkers, and clinical characteristics with a *p*-value < 0.10 at univariable analysis were selected for the multivariable Cox regression analysis. Again, only those with a *p*-value < 0.10 were maintained in the final multivariable model. The final model was derived using a stepwise backward procedure based on the likelihood ratio test. Hazard ratios (HR) for Cox regression models were reported together with a 95% confidence interval (CI) and *p*-value. The interplay between systemic inflammatory indexes and FDG PET-derived parameters was evaluated according to the methodology previously proposed by Castello et al. [22] and by Bauckneht et al. [59].

All the above-described analyses were repeated, adding to the model the degree of each parameter’s variation at the radiological progression with respect to baseline (measured as the ratio between the time of radiological progression with respect to baseline, and termed “ratio”) and classes of response according to PERCIST, irRC, and iRECIST criteria.

All tests are two-sided. Analyses were conducted with IBM-SPSS vers. 23.

## 5. Conclusions

Identifying prognostic indicators of progression early in the course of treatment is crucial for risk stratification and subsequent improvement of patients’ management (including selection for different or combined therapies). The degree of systemic inflammation, the quantification of the metabolically active tumor burden, and their combination could be used to early disclose the radiological progression in NSCLC patients receiving ICI. Further studies in a larger group of patients are needed to confirm this evidence and extend it to the pretreatment setting, thus potentially improving patients’ selection for immunotherapy treatment.

## Figures and Tables

**Table 1:** Clinical characteristics of enrolled patients at the time of radiological progression.

Age		70.6 (Range 50.3–81.5)
Gender	Female	16/45 (35.5%)
	Male	29/45 (64.5%)
ECOG PS	0	18/45 (40%)
	1	25/45 (55.5%)
	2	2/45 (4.5%)
Steroid use	Yes No Unknown	13/45 (29%) 21/45 (47%) 11/45 (24%)
Presence of brain metastases	Yes No	5/45 (11%) 40/45 (89%)
Smoking status	Never smoker	5/45 (11%)
	Former smoker	29/45 (65%)
	Smoker	11/45 (24%)
Histology	Squamous	11/45 (24%)
	Non-squamous	34/45 (76%)
Prior surgery	Yes	17/45 (38%)
	No	28/45 (62%)
Prior lines of therapy	1	18/45 (40%)
	2	16/45 (36%)
	≥3	11/45 (24%)
Number administered cycles of ICI before PD		6.6 (range 4–33)

**Table 2:** Clinical characteristics, systemic inflammation, and FDG PET/CT parameters at radiological progression.

	Univariate Analysis		Multivariate Analysis	
	HR (95% CI)	*p* Value	HR (95% CI)	*p* Value
Clinical characteristics				
ECOG Performance Status		0.149		
0–1	1.00 (ref)	-		
2	1.858 (0.80–4.31)	-		
Presence of brain metastases		0.823		
No	1.00 (ref)	-		
Yes	1.104 (0.46–2.63)	-		
Steroids use		0.484		
No	1.00 (ref)	-		
Yes	1.335 (0.59–2.99)	-		
Inflammatory biomarkers				
NLR (1-unit)	1.089 (1.02–1.16)	0.013		
d-NLR (1-unit)	1.206 (1.04–1.39)	0.013		
LMR (1-unit)	1.031 (0.89–1.19)	0.684		
PLR (100-unit)	1.000 (0.99–1.002)	0.771		
SII (100-unit)	1.002 (1.001–1.004)	<0.0001	1.002 (1.001–1.002)	<0.0001
FDG-PET parameters				
SUVmax (1-unit)	1.032 (0.98–1.07)	0.161		
MTV (1-unit)	1.001 (1.001–1.002)	<0.0001	1.001 (1.001–1.002)	<0.0001
TLG (1-unit)	1.001 (1.001–1.002)	<0.0001		

**Table 3:** Systemic inflammation and FDG PET/CT parameters in the evaluation of response.

	Univariate Analysis		Multivariate Analysis	
	HR (95% CI)	*p* Value	HR (95% CI)	*p* Value
CT-based response criteria				
irRC classes		0.005		0.027
PR	1.00 (ref)	-	1.00 (ref)	-
SD	4.826 (0.55–42.01)	-	3.746 (0.42–33.04)	-
PD (uPD + cPD)	10.573 (1.28–87.22)	-	7.742 (0.91–65.49)	-
iRECIST classes		0.024		
iPR	1.00 (ref)	-		
iSD	5.088 (0.31–86.38)	-		
iPD (iuPD + icPD)	7.887 (1.03–60.55)	-		
Inflammatory biomarkers				
NLRratio	1.080 (0.96–1.20)	0.164		
dNLRratio	1.083 (0.97–1.21)	0.140		
LMRratio	1.057 (0.97–1.14)	0.164		
PLRratio	0.873 (0.48–1.56)	0.648		
SIIratio	1.186 (1.03–1.36)	0.019	1.162 (0.98–1.37)	0.041
FDG-PET parameters				
PERCIST classes		0.352		
PMR	1.00 (ref)	-		
SMD	2.232 (0.52–9.46)	-		
PMD	1.482 (0.31–7.01)	-		
SUVmax-ratio	3.285 (1.25–8.62)	0.016		
MTVratio	1.217 (1.08–1.36)	<0.001		
TLGratio	1.209 (1.21–1.34)	<0.001	1.171 (1.04–1.31)	0.007

**Table 4:** IMPI and IMPIR are independent predictors of OS.

	Multivariate Analysis	
	HR (95% CI)	*p* Value
IMPI		0.0004
Low risk	1.00 (ref)	-
Intermediate risk	2.271 (0.99–5.19)	-
High risk	7.036 (2.55–19.40)	-
IMPIR		0.003
Low risk	1.00 (ref)	-
Intermediate risk	1.204 (0.52–2.78)	-
High risk	6.259 (2.16–18.14)	-

## Data Availability

The data that support the findings of this study are available on request from the corresponding author.

## Supplementary Materials

The following are available online at https://www.mdpi.com/article/10.3390/cancers13133117/s1, Figure S1: IMPI and IMPIR calculation.
