# Supplementary material for: The Role of the Immune Metabolic Prognostic Index in Patients with Non-Small Cell Lung Cancer (NSCLC) in Radiological Progression during Treatment with Nivolumab
Source: Cancers (Basel). 2021 Jun 22;13(13):3117. doi: 10.3390/cancers13133117 (PMC8268031; doi:10.3390/cancers13133117)
Supplement: Supplementary file 1 [file cancers-13-03117-s001.zip › cancers-1268635-supplementary.pdf]

cancers

Article

Supplementary Material: The Role of the Immune Metabolic Prognostic Index in Patients with Non-Small Cell Lung Cancer (NSCLC) in Radiological Progression during Treatment with Nivolumab

Matteo Bauckneht [1,*], Carlo Genova [2,3], Giovanni Rossi [2], Erika Rijavec [4], Maria Giovanna Dal Bello [2], Giulia Ferrarazzo [1], Marco Tagliamento [2], Maria Isabella Donegani [1,5], Federica Biello [6], Silvia Chiola [1,5], Lodovica Zullo [7], Stefano Raffa [1,5], Francesco Lanfranchi [1,5], Giuseppe Cittadini [8], Cecilia Marini [5,9], Egesta Lopci [10], Gianmario Sambuceti [1,5], Francesco Grossi [4] and Silvia Morbelli [1,5]

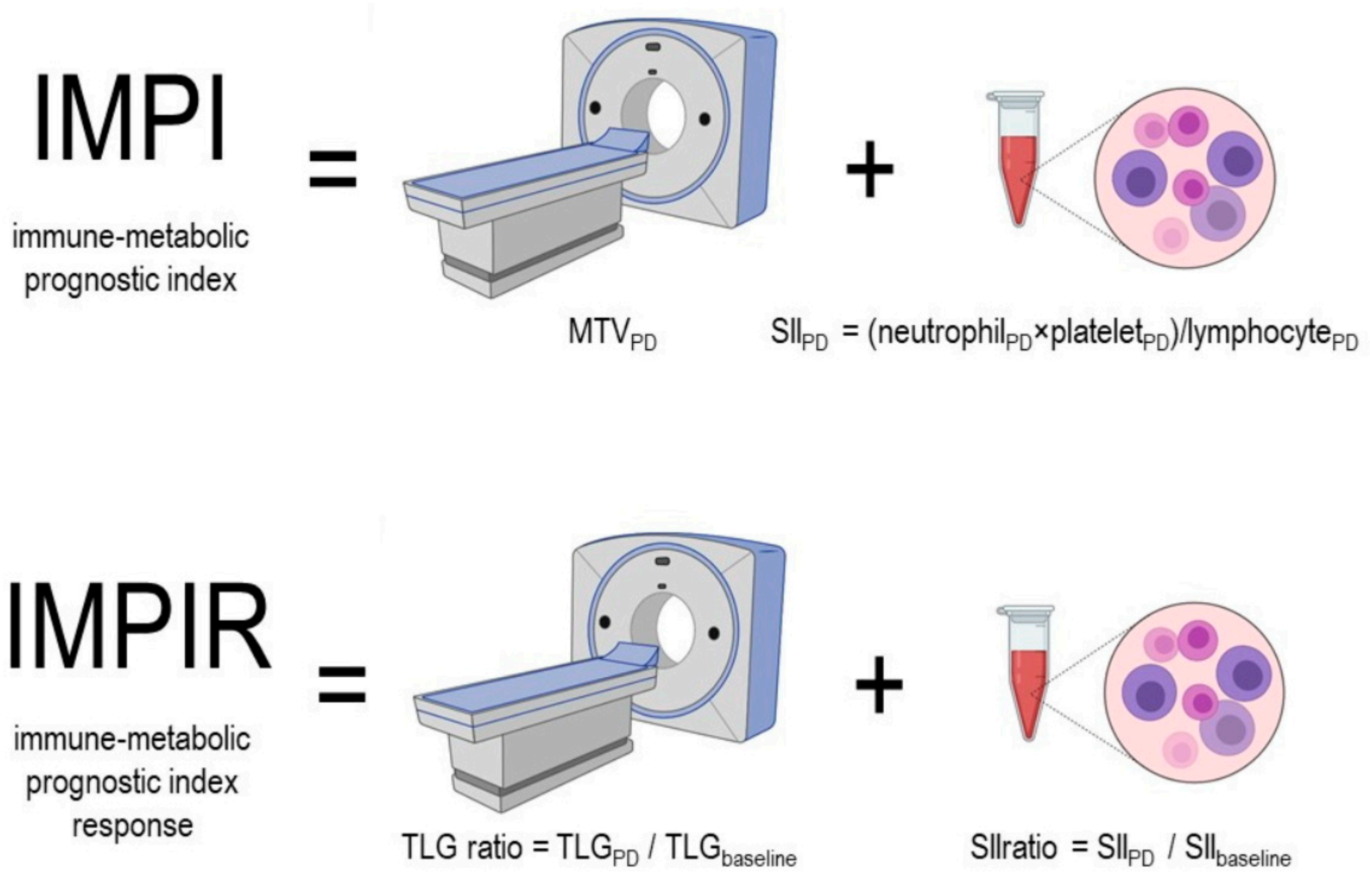

Figure S1. IMPI and IMPIR calculation.
